# Determining gene response patterns of time series gene expression data using R

**DOI:** 10.1186/1471-2105-15-S10-P24

**Published:** 2014-09-29

**Authors:** Kevin L O’kello, Vinhthuy Phan

**Affiliations:** 1 Bioinformatics Program, University of Memphis, Memphis TN 38152, USA

## Background

The rapid advancement of sequencing technologies has not been without challenges. The extensive size of genomes as well as the need to comprehend the vast complexities of genomic information has fostered the need to utilize more robust computational methods and statistical analysis tools. In a gene expression study involving multiple treatments, a time series analysis of differentially expressed genes provides great insights into the replicates. Such information is useful in identifying the potential sources of variation that cannot be easily extrapolated from a generalized experimental approach. It is also essential to be able to group the identified gene expression patterns in order to attain meaningful interpretations.

## Materials and methods

The experiment employs the use of R software to identify gene expression patterns in a dataset involving parathyroid tumor treatments [1]. The treatments were generated in a three day time interval. The R tool provides important packages that are ideal for gene expression studies and also delivers significant visualization and inference resources [2]. The proposed approach for this analysis consists of two stages. First, the Kruskal-Wallis test is used to identify differentially expressed genes. Second, patterns of differentially expressed genes are determined using the Wilcoxon rank-sum test.
